# Heavy metals analysis, phytochemical, phytotoxic and anthelmintic investigations of crude methanolic extract, subsequent fractions and crude saponins from *Polygonum hydropiper* L

**DOI:** 10.1186/1472-6882-14-465

**Published:** 2014-12-03

**Authors:** Muhammad Ayaz, Muhammad Junaid, Fazal Subhan, Farhat Ullah, Abdul Sadiq, Sajjad Ahmad, Muhammad Imran, Zul Kamal, Sajid Hussain, Syed Majid Shah

**Affiliations:** Department of Pharmacy, University of Malakand, Khyber Pakhtoonkhwa, 18000, KPK Pakistan; Department of Pharmacy, University of Peshawar, Peshawar, Pakistan; Department of Pharmacy, Shaheed Benazir Bhutto University, Sheringal (Dir Upper), Kohat, KPK, Pakistan; Department of Pharmacy, Kohat University of Science and Technology (KUST) Kohat, Kohat, Pakistan

**Keywords:** *Polygonum hydropiper*, Phytotoxic, Anthelmintic, Heavy metals and saponins

## Abstract

**Background:**

*Polygonum hydropiper* L decoctions are traditionally used in the treatment of various ailments including inflammation, dyspepsia, diarrhea, menorrhagia, hemorrhoids, helminthiasis and CNS disorders. Present study was undertaken to investigate *P. hydropiper* L. for heavy metals content, phytoconstituents, Phytotoxic and anthelmintic activities to explore its toxicological and pharmacological potentials and rationalize its ethnomedicinal uses.

**Methods:**

Plant crude powder, methanolic extract, fractions and soil samples were analyzed for heavy metals using atomic absorption spectrophotometer. Qualitative phytochemical analysis of the plant extracts was carried out for the existence of alkaloids, flavonoids, glycosides, anthraquinones, saponins, terpenoids, sterols and tannins. Radish seeds phytotoxicity assay was used to study phytotoxic action of plant extracts. *Pheretima posthuma* and *Ascaridia galli* were used to study anthelmintic potential of the plant using albendazole and levamisole HCl as standard drugs.

**Results:**

Plant crude powder, methanolic extract (Ph.Cr), its subsequent fractions; *n*-hexane (Ph.Hex), chloroform (Ph.Chf), ethyl acetate (Ph.EtAc), *n*-Butanol (Ph.Bt), aqueous (Ph.Aq), saponins (Ph.Sp) and soil samples were found to contain copper (Cu), iron (Fe), chromium (Cr), zinc (Zn), lead (Pb), nickel (Ni), cadmium (Cd) and lead (Pb) in different concentrations. In crude powder of the plant, heavy metals concentrations were within WHO specified limits, whereas different fractions and soil samples exhibited high metals content. Ph.Cr was tested positive for the presence of alkaloids, flavonoids, saponins, tannins, triterpenoids and anthraquinone glycosides. Among different fractions Ph.EtAc, Ph.Sp, Ph.Chf and Ph.Bt were most effective causing 89.32, 89.25, 86.68 and 85.32% inhibition of seeds in phytotoxicity assay, with IC_50_ values of 50, 60, 35 and 100 μg/ml respectively. In anthelmintic study, Ph.Sp, Ph.Chf, Ph.EtAc and Ph.Cr were most effective against *P. posthuma* at 10 mg/ml concentration with an average death time of 50, 64.67, 68.67 and 71 minutes respectively. Ph.EtAc, Ph.Chf and Ph.Aq were most effective against *A. galli* with average death time of 7, 9 and 10 min respectively at 1 mg/ml concentration.

**Conclusions:**

Our findings indicate that *P. hydropiper* contains different heavy metals and secondary metabolites. Different fractions exhibited phytotoxic and anthelmintic activites comparable to control drugs, thus provide pharmacological basis for ethnomedicinal uses of this plant.

## Background

Pakistan is naturally gifted with a wide variety of medicinal plants due to its diverse climate and edaphic conditions. At least six thousand flowering plants have been reported currently from Pakistan among which 400–600 are of high medicinal value [1]. Even though a large number of clinically effective drugs have been developed, indigenous phytotherapy is still practiced in several developing countries [2]. About 85% of primary health care medications are obtained from natural sources worldwide [3]. It is desirable to utilize the therapeutic potential of higher plants to get novel, affordable, more effective and safer natural drugs.

*Polygonum hydropiper,* also known as "smartweed" belong to *Polygonaceae*, a family comprising of about 50 genera and 1200 species [4]. Domestically the plant is used as anti-inflammatory, carminative, astringent, diuretic, CNS stimulant, diaphoretic, stomachic, emmenagogue, anthelmintic, in bleeding disorders and in diarrhea [5]. Conventionally, the whole plant decoction is used to treat different diseases like dyspepsia, menorrhagia, hemorrhoids, and skin itching [6]. Ethnomedicinally *P. hydropiper* and other species of *Polygonaceae* are used to treat fever, chill, joint pain, oedema and infectious diseases for more than 300 years in Chinese folk medicine [7]. Moreover it is also used to treat rheumatoid arthritis [8], Prostate gland inflammation, diarrhea [9], insomnia, kidney diseases, hemorrhoids [10], hypertension, angina and other cardiovascular diseases [11, 12]. We recently reported *P. hydropiper* for antioxidant, anticholinestrase activities and its potential use in the treatment of neurodegenerative diseases [13]. Other species of *Polygonaceae* including *Polygonum persicaria* and *Polygonum plebejum* are reported for cytotoxic and phytotoxic activities [14].

Though plant based therapies are known to be free from side effects in comparison to allopathic drugs, still the presence of toxic heavy metals in such products can cause serious health consequences [15, 16]. Recent scientific research has revealed that several plants used as food or in traditional medicine are potentially toxic, mutagenic and carcinogenic [17, 18]. World Health Organization (WHO) recommends that such products must be evaluated for the extent of heavy metals prior to use in human beings.

Phytochemicals are broadly grouped into two categories namely, primary constituents and secondary metabolites. Primary constituents include amino acids, proteins, common sugars and chlorophyll whereas secondary constituents include alkaloids, phenolic compounds, flavonoids, saponins, essential oils, tannins and terpenoids [19]. Majority of phytochemicals have been known to bear valuable therapeutic activities and the plants thus find their medicinal value due to respective phytochmical constituents they contain. Saponins, glycosides widely distributed in the plants kingdom, consist of a diverse group of compounds characterized by their structure containing a steroidal aglycone and one or more sugar chains. Their structural diversity is reflected in their physicochemical and biological properties, which are exploited in a number of traditional, industrial [20] and pharmacological uses including, antimicrobial, antidiabetic, cytotoxic, antispasmodic, antioxidant, antineoplastic and anthelmintic [21–25].

Globally there are two billion people of different ages which are parasitic worms carriers [26]. These helminthes cause malnutrition, malabsorption, iron-deficiency anemia and obstruction of small intestine which leads to impairment in physical growth cognitive and intellectual potential of children [27, 28]. Thus treatment of individual infected patients and awareness in the community is very important [29]. Beside this, helminthiasis is a common animals disease in developing countries, leading to reduction in the production of milk [30]. The development of novel anthelmintic drugs is not very rapid in the developing countries due to less financial benefits in comparison to investment. Beside this, emergence of helminthes resistance against anthelmintic drugs [31] has led to the proposal of screening medicinal plants for their anthelmintic activity. So there is need to develop new, cost-effective drugs for the treatment of parasitic infections [32]. Plants are potential sources of anthelmintic drugs and a variety of medicinal plants have been employed to treat parasitic infections in man and animals [33, 34]. Heavy metals are toxic for human beings and are also phytotoxic in nature. Thus aims of the current study were heavy metals analysis in different fractions and their relation with phytotoxicity. Further we also investigated secondary metabolites and antelmintic potential of *P.hydropiper* extracts and crude saponins.

## Methods

### Chemicals and drugs

Albendazole and Levamisole HCl used as standard drugs in anthelmintic activity were purchased from GlaxoSmithKline and ICI Pakistan respectively. Paraquat (Sigma-Aldrich EC 231-791-2) was used as positive control in phytotoxic assay. Solvents used in extraction, metals analysis and reagents for phytochemical tests were of pure analytical grade.

### Plant material, extraction and fractionation

*P. hydropiper* whole plant was collected from Talash Valley, Khyber Pakhtoonkhwa Pakistan in July, 2013 and was identified by Dr. Gul Rahim, Arid Agriculture University, Rawalpindi, Pakistan. A voucher (H.UOM.BG.107) of the specimen was deposited at the herbarium of University of Malakand, Chakdara (Dir), Pakistan. Plant material was cleansed with distilled water and was shade dried for 30 days. Thereafter, it was coarsely crushed using cutter mill. The powder material (4.5 kg) was soaked in 80% methanol (22 L) in large container for 15 days with frequent shaking. Extraction with methanol was repeated three times, added to original extract and filtered through muslin cloth and then through filter paper [35]. The filtrate was concentrated using rotary evaporator (Heidolph Laborota 4000, Schwabach, Germany) under reduced pressure at 40°C resulting in 290 g (6.44%) of dark brown semisolid mass. Crude methanolic extract (250 g) of *P. hydropiper* (Ph-Cr) was suspended in 500 ml of distilled water and consequently partitioned with *n*-hexane (3 × 500 ml), chloroform (3 × 500 ml), ethyl acetate (3 × 500 ml), *n*-butanol (3 × 500 ml) and finally aqueous fraction was left. Extraction yield were 68 g (27.2%) for Ph.Hex, 27 g (10.8%) for Ph.Chf, 13 g (5.2%) for Ph.EtAc, 11 g (4.4%) for Ph.Bt & 37 g (14.8%) for Ph.Aq fractions.

### Extraction of crude saponins

Plant material (powdered) weighing 60 g was transferred to a conical flask and was soaked with 100 ml of 20% ethanol. The mixture was heated for 4 h at 55°C using water bath and constant shaking. Thereafter, it was filtered and was again extracted with 200 ml of 20% ethanol. Volume of the liquid extracted was reduced to 40 ml using water bath and transferred it to a separating funnel. Diethyl ether (20 ml) was added to it with vigorous shaking to separate the two phases (Diethyl ether and water). Organic layer was discarded, whereas 60 ml of *n*-butanol was added to aqueous fraction. The combined aqueous - butanol mixture was washed two times with 5% NaCl solution. Finally solvents were evaporated using water bath to get saponins (9 g) with a percent yield of 15% [36].

### Metals analysis

For metal analysis chemicals of analytical grade were used to prepare sample of the crude drug and different fractions. From each fraction a sample weighing 2 g was taken in a crucible and was ignited in a muffle furnace at 550°C for 6 h. The ash produced was digested in 5 ml of concentrated nitric acid followed by evaporation on hot plate. Small amount of distilled water was added to the digested residue, filtered and volume was made to 30 ml using distilled water. The solutions formed were quantitatively analyzed using atomic absorption spectrophotometer (model 1100; Perkin Elmer, Waltham, Massachusetts, USA) for the heavy metals including, iron (Fe), lead (Pb), copper (Cu), cadmium (Cd), zinc (Zn), nickel (Ni) and chromium (Cr) [37].

### Phytochemical investigation

Qualitative phytochemical analysis of the plant extracts was carried out for the existence of alkaloids, flavonoids, glycosides, anthraquinones, saponins, terpenoids, sterols and tannins using the method reported previously [38]. The presence of alkaloids was determined using Dragendorff’s reagent. Liebermann Burchard test was used for the detection of steroids and triterpenoids. Briefly Ph.Cr was mixed with few drops of acetic anhydride followed by boiling and cooling. Concentrated H_2_SO_4_ was then added from the sides of the test tube and was observed for the formation of a brown ring at the junction of two layers. Formation of green color at the upper layer and deep red color at the lower layer indicate presence of steroids and triterpenoids respectively. For the detection of glycosides "Keller Killiani" Test was used. Test solution was added a few drops of glacial acetic acid and Ferric chloride solution and were properly mixed. A few drops of concentrated H_2_SO_4_ were added to this mixture and observed for the formation of two layers. Lower reddish brown layer and upper acetic acid layer which turns bluish green indicates positive test for glycosides. Presence of saponins was detected based on the formation of froth upon vigorous shaking using diluted samples. Anthraquinones were detected by boiling the test sample with 1 ml of H_2_SO_4_ in a test tube followed by filtration. After cooling the filtrate was shacked with equal volume of chloroform and lower layer was separated and shacked with dilute ammonia. Formation of rose pink to red color ammonical layer indicate presence of anthraquinone glycosides. Ph.Cr was treated with gelatin solution and the appearance of white precipitate was observed which indicate the presence of tannins.

### Phytotoxicity assay

Radish seeds were used to study phytotoxic potential of plant extracts employing method previously described [39]. Mercuric chloride **(**HgCl_2_) 0.1% solution was prepared in distilled water and radish seeds were put in it to be surface sterilized for 2–3 minutes. The seeds were rinsed with autoclaved distilled water and were dried using sterilized blotting paper. 0.5 ml of each sample solution was transferred to sterilized Petri dish containing Whatman paper and methanol was vacuum evaporated. Methanol (5 ml per Petri dish) was used as negative control (blank). To each test group Petri dish 5 ml of distilled water was added, and 25 radish seeds were placed in each plate at sufficient distance using sterilized forceps. All plates were incubated at 25°C in dim light. After 3–5 days of incubation, number of seeds germination and percent inhibition of root length was calculated using formula:


The test was performed in triplicate and data were analyzed by ANOVA.

### Anthelmintic assay

Adult earthworms (*Pheretima posthuma* L. Vaill) and roundworms (*Ascaridia galli)* were used to investigate anthelmintic potential of Ph.Cr, Ph.Hex, Ph.Chf, Ph.EtAc, Ph.Bt, Ph.Aq and Ph.Sp using method previously described [40]. Earthworms were selected for *in-vitro* investigation due to their high physiological and anatomical resemblance with human intestinal roundworm *Ascaris lumbricoides* [33, 34] *.* The earth worms were collected from marshy soil having small pellets on the surface in the locality of University of Malakand, Chakdara Khyber Pakhtoonkhwa, Pakistan, with an average length of 8-9 cm. For collection of round worms intestines of freshly slaughtered fowls were dissected and were maintained in normal saline solution. average length of roundworms was 5-7 cm. Different dilutions (1, 5 and 10 mg/ml) of plant extracts, Ph.Sp and standard drugs (albendazole and levamisole HCl) were prepared. From these solutions, 25 ml each were transferred to sterile Petri dishes and six worms were added to each Petri dish with the help of spatula. Time for complete paralysis of the worms, when no movement (except with vigorous shaking) was observed. Similarly, death time was noted when no movement was observed even with vigorous shaking and exposure to hot water at 50°C temperature.

### Statistical analysis

One-way ANOVA followed by Dunnett’s multiple comparison test were applied for the comparison of positive control with the test groups. *P* values less than or equal to 0.05 were considered statistically significant. Graph Pad Prism and XL sheet were used to draw the curves and IC_50_ values. The standard error of mean (SEM) were calculated at 95% confidence intervals.

## Results

### Metals analysis

The concentrations of various metals in dry powder, Ph.Cr and different fractions i.e. Ph.Hex, Ph.Chf, Ph.EtAc, Ph.Bt, Ph.Aq, Ph.Sp of *P. hydropiper* are summarized in Table 1. Results indicate that in crude powder of *P. hydropiper,* concentrations of all heavy metals including Cu, Fe, Cr, Zn, Ni and Cd were 7.21 ± 0.23, 95.00 ± 0.55, 1.51 ± 0.45, 7 ± 0.06, 0.35 ± 0.154 and 0.199 ± 0.001 ppm while the concentration of Pb in crude powder was below the detectable range. On the other hand, concentrations of these metals were high in soil samples as compared with the Ph.Cr and different fractions extracted from crude extract. Cr concentration was below detection limit in almost all fractions except Ph.Cr.

**Table 1:** **Metal contents (ppm) in crude powder, soil and different fractions of** ***P. hydropiper***

Sample	Cu	Fe	Cr	Zn	Ni	Cd	Pb
Crude Powder	7.21 ± 0.23	95.00 ± 0.55	1.51 ± 0.45	7 ± 0.06	0.199 ± 0.001	0.35 ± 0.154	Nd
Ph.Cr	41.16 ± 0.06	10.89 ± 0.001	243.12 ± 0.31	Nd	Nd	22.85 ± 0.05	79.37 ± 1.47
Ph.Hex	45.96 ± 0.07	76.15 ± 0.27	Nd	4.32 ± 0.009	11.45 ± 0.02	20.28 ± 0.02	67.30 ± 0.31
Ph.Chf	134.70 ± 0.15	155.88 ± 0.21	Nd	Nd	7.05 ± 0.01	16.02 ± 0.11	55.44 ± 2.03
Ph.EtAc	25.60 ± 0.25	26.55 ± 0.09	Nd	Nd	9.56 ± 0.08	9.55 ± 0.02	Nd
Ph.Bt	51.40 ± 0.03	135 ± 0.70	Nd	28.28 ± 0.01	Nd	37.1 ± 0.013	70.3 ± 1.57
Ph.Aq	34.60 ± 0.05	124.65 ± 0.04	Nd	139.92 ± 0.002	Nd	23.18 ± 0.02	Nd
Soil	125.30 ± 0.50	113 ± 1.54	76.95 ± 0.59	89.34 ± 0.46	19 ± 1.25	55.23 ± 1.05	90.15 ± 0.56

**Cu:** copper; **Fe:** iron; **Cr:** chromium; **Zn:** zinc; **Pb:** lead; **Ni:** nickel **Cd:** cadmium; **Pb:** lead.

**Nd:** Not detected/ below detectable concentrations.

### Phytochemical analysis

Ph.Cr was tested positive for the presence of alkaloids, flavonoids, saponins, tannins, triterpenoids anthraquinones, glycosides, while tested negative for the presence of steroids (Table 2).

**Table 2:** **Phytochemical constituents in crude extract of** ***P. hydropiper***

S. no	Phytochemical class	Test performed	Observations	Results
**1**	Alkaloids	Dragendorff’s Test	Turbidity/precipitation	**+**
**2**	Flavonoids	Ferric chloride test	Formation of yellow color which changed to colorless on acid addition	**+**
**3**	Saponins	Froth Test	1. Stable froth formation	**+**
			2. Emulsion formation after olive oil	**+**
**4**	Tannins	Gelatin Test	appearance of white precipitate	**+**
**5**	Steroids	Liebermann Burchard test	green to pink color was absent	**-**
**6**	Glycosides	Keller Killiani	Lower reddish brown layer & upper acetic acid layer which turns bluish green	**+**
**7**	Anthraquinones	Bontrager’s test	Formation of red, violet or pink color f in aqueous layer	**+**
**8**	Triterpenoids	Liebermann Burchard test	Appearance of reddish brown-deep red color	**+**

**+**; Phytoconstituent present **-**; Phytoconstituent Absent.

### Phytotoxic assay

The crude extract and its fractions showed concentration dependent phytotoxicity. Regarding seeds inhibition, Ph.Chf, Ph.EtAc and Ph.Sp were most potent causing 86.68 ± 3.5%, 89.32 ± 4.8% and 89.25 ± 2.2% inhibitory effects on seeds germination at 1 mg/ml concentration with IC_50_ values of 35, 50 and 60 μg/ml, respectively. This was followed by Ph.Cr, Ph.Hex, Ph.Bt and Ph.Aq fractions which showed 68.00 ± 2.8% (IC_50_ 310 μg/ml), 77.32 ± 1.3% (IC_50_ 215 μg/ml), 85.32 ± 3.5% (IC_50_ 100 μg/ml), 73.32 ± 3.5% (IC_50_ 485 μg/ml) inhibition of seed germination respectively at 1 mg/ml concentration. Ph.Bt, Ph.Sp, Ph.EtAc and Ph.Cr showed highest inhibitory effect on root length causing 86.26 ± 1.25% (IC_50_ 8 μg/ml), 91.90 ± 0.75% (IC_50_ 10 μg/ml), 92.34 ± 1.30% (IC_50_ 11 μg/ml) and 82.38 ± 0.68% (IC_50_ 11 μg/ml) respectively, at 1 mg/ml concentration (Table 3).

**Table 3:** **Phytotoxic effect of the crude extract, subsequent fractions and crude saponins of** ***P. hydropiper*** **against radish seeds**

Samples	Conc. mg/ml	Average root length (mm) mean ± SEM	Average no of seeds inhibited mean ± SEM	Root length inhibition % mean ± SEM	IC _50_ μg/ml	Seeds inhibition % mean ± SEM	IC _50_ μg/ml
**Ph.Cr**	1000	5.13 ± 0.20	17.00 ± 0.58	82.38 ± 0.68***	16	68.00 ± 2.8***	310
	500	6.87 ± 0.26	14.66 ± 0.89	76.41 ± 0.89***		58.64 ± 3.5***	
	250	8.23 ± 0.20	11.33 ± 0.89	71.74 ± 0.68***		45.32 ± 2.8***	
**Ph.Hex**	1000	5.33 ± 0.49	19.33 ± 0.33	82.70 ± 1.6***		77.32 ± 1.3***	
	500	5.03 ± 0.23	17.33 ± 0.67	81.73 ± 0.78***	124	69.32 ± 2.7***	215
	250	12.03 ± 0.48	12.33 ± 0.67	58.70 ± 1.60***		49.32 ± 2.7***	
**Ph.Chf**	1000	3.9 ± 0.52	21.67 ± 0.89	86.61 ± 1.70***		86.68 ± 3.5***	
	500	8.4 ± 0.33	19.00 ± 1.15	71.16 ± 1.10***	145	76.00 ± 4.6***	35
	250	11.3 ± 0.43	18.00 ± 0.00	61.20 ± 1.40***		72.00 ± 0.0***	
**Ph.EtAc**	1000	2.23 ± 0.40	22.33 ± 1.20	92.34 ± 1.30^ns^		89.32 ± 4.8***	
	500	4.9 ± 0.43	21.66 ± 1.20	83.17 ± 1.40***	11	85.32 ± 4.8 ^ns^	50
	250	5.9 ± 0.20	17.67 ± 0.89	79.74 ± 0.68*		70.68 ± 3.5***	
**Ph.Bt**	1000	4.0 ± 0.37	21.33 ± 0.89	86.26 ± 1.25***		85.32 ± 3.5***	
	500	5.93 ± 0.49	18.00 ± 0.58	79.64 ± 1.60***	8	72.00 ± 0.0***	100
	250	7.03 ± 0.23	16.00 ± 0.00	75.86 ± 0.78***		64.00 ± 0.0***	
**Ph.Aq**	1000	6.2 ± 0.23	18.33 ± 0.89	78.71 ± 0.78***		73.32 ± 3.5***	
	500	8.8 ± 0.37	12.67 ± 0.89	69.79 ± 1.20***	170	50.68 ± 3.5***	485
	250	12.9 ± 0.48	07.00 ± 1.15	55.71 ± 1.60***		28.00 ± 4.6***	
**Ph.Sp**	1000	5.00 ± 0.50	22. 00 ± 1.10	91.90 ± 0.75 ^ns^		89.25 ± 2.2***	
	500	7.25 ± 0.20	19.00 ± 1.50	82.10 ± 1.50***	10	77.25 ± 1.0***	60
	250	8.50 ± 0.58	17.00 ± 0.89	79.74 ± 0.68*		70.68 ± 1.5***	
**Positive control**	1000	1.93 ± 0.49	24.67 ± 0.89	93.64 ± 1.60		98.61 ± 1.70	
	500	3.03 ± 0.23	22.16 ± 1.10	89.20 ± 1.40	0.89	88.22 ± 0.23	0.89
	250	5.71 ± 0.78	21.90 ± 0.52	83.33 ± 1.20		87.93 ± 0.49	
**Negative control**	———	29.13 ± 0.37	———	——	——	——	——

Standard drug (P. Control) Paraquat IC_50_ = 0.89 μg/ml. Results expressed as average seeds inhibition, % inhibition and Inhibition of root length (mean ± SEM n = 3) and IC_50_. Values significantly different as compare to Standard drug; *P < 0.05, *** P < 0.001.

### Anthelmintic assay

**Figure 1:**
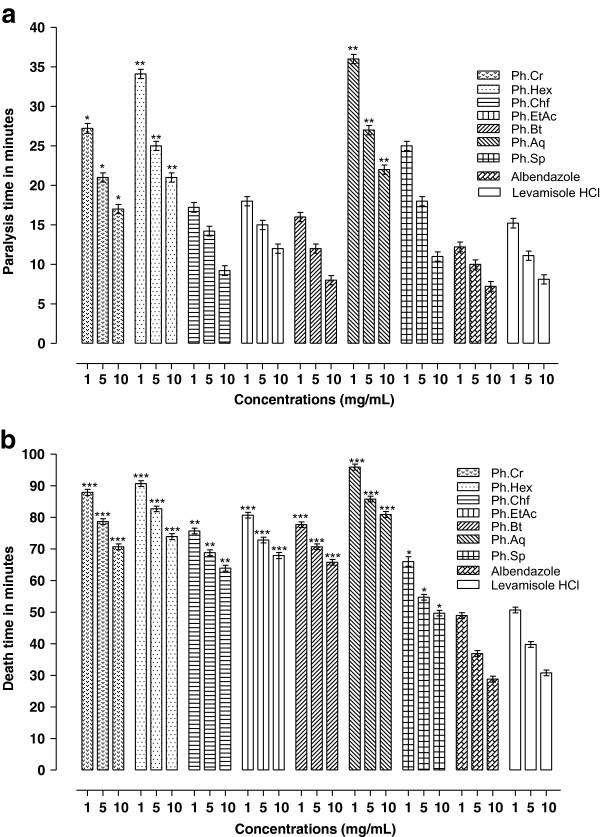
**Anthelmintic activity of Ph.Cr, subsequent fractions and Ph.Sp of** ***P. hydropiper*** **against** ***P. posthuma*** **(a: Paralysis time b: Death time in minutes).**

**Figure 2:**
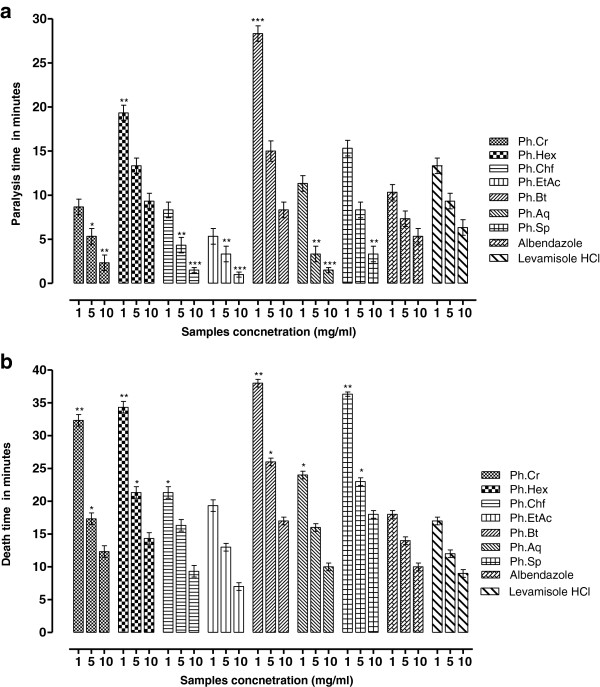
**Anthelmintic activity Ph.Cr, subsequent fractions and Ph.Sp of** ***P. hydropiper*** **against** ***Ascaridia galli*** **(a: Paralysis time b: Death time in minutes).**

Results of *P. hydropiper* anthelmintic activity against *P. posthuma* and *A.galli* are given in Figures 1(a,b) and 2 (a,b). Among different fractions Ph.Sp, Ph.Chf, Ph.Bt and Ph.EtAc were most effective against *P. posthuma* with death time of 50.00 ± 0.75, 64.67 ± 2.51, 66.33 ± 1.53 and 68.67 ± 1.52 minutes respectively at 10 mg/ml concentration. Whereas the death times of *P. posthuma* against standard drugs albendazole and levamisole HCl were 29.33 ± 1.54 and 31.33 ± 2.08 minutes, respectively, at 10 mg/ml concentration. Death time for Ph.Cr, Ph.Hex, Ph.Aq were 71.00 ± 1.73, 74.67 ± 1.52 and 81.67 ± 0.57 minutes, respectively, at 10 mg/ml concentration. Paralysis time for different fractions against *P. posthuma* were; Ph.Cr (17.00 ± 1.00), Ph.Hex (21.00 ± 3.00), Ph.Chf (9.67 ± 2.51), Ph.EtAc (12.67 ± 0.57), Ph.Bt (8.33 ± 1.53), Ph.Aq (22.67 ± 2.51) and Ph.Sp (11.00 ± 0.58) minutes. While for albendazole and levamisole HCl the paralysis times were 7.67 ± 0.57 and 8.33 ± 0.58 minutes respectively at 10 mg/ml concentration. In anthelmintic activity against *A. galli*, Ph.EtAc, Ph.Chf and Ph.Aq were most effective with average death time of 7, 9 and 10 minutes respectively at 1 mg/ml concentration. Activity of these fractions were comparable to control drugs albendazole and levamisole HCl which killed all tested roundworms in 10 and 9 minutes respectively at the same concentration. All other fractions were effective in concentration dependent manner (Figure 2a,b).

## Discussion

High levels of heavy metals, like lead, cadmium, chromium and manganese which are present in soil and waste water utilized for agricultural irrigation, can be accumulated into these systems. Because of their non-biodegradability features, toxicity effects onto several crops and consequently their bioavailability in human beings may be hazardous. Presence of heavy metals and their interactions with essential trace elements can cause serious health consequences. WHO recommends that plant materials, which are used to make finished products for use, may be checked for the occurrence of heavy metals contamination. Pb and Cd are non-essential trace elements having no useful function in the body. Pb poisoning causes convulsions, chronic nephritis, central nervous system disorders and colic whereas Cd after chronic accumulation in the body causes liver and kidney damages. The consequences of chronic Cr intake include skin rash, nasal itch, kidney and liver damage, bleeds, stomach upsets and lungs cancer [41]. According to WHO, the specific limit of heavy metals Pb, Cd, Cr, Cu, Ni and Zn in medicinal plants and food are 10, 1, 1.5, 10, 1.5 and 50 ppm, respectively [42, 43]. In the present study, we observed that crude powder of *P. hydropiper* contains the aforementioned metals in lower concentrations than the permissibility. But the levels of heavy metals particularly Pb, Cd and Cr were high in different fractions. As fractions are extracted from greater quantity (Kg) of crude powder so high concentrations of these metals are concentrated in these fractions. High concentrations of these metals can be attributed to their concentration in the soil and marshy place from where the plant was collected.

Different weeds are amongst the most key factors responsible for significant reduction in crops yield. In Sindh and Punjab provinces of Pakistan almost forty weed species have been reported which account for about 40% loss in wheat crop yield on the average. To counteract these unwanted weeds different chemicals are used. However, such chemicals are frequently associated with environmental pollution, residual toxicity, carcinogenesis, high cost and hence their use is restricted [42, 44]. Consequently, the search for alternative herbicides which are safe and cost-effective is needed. Natural herbicides may be among these alternatives. In the current phytotoxic assay, Ph.Chf was most potent and it is also obvious from the results that this fraction contain high concentration of heavy metals. So it may be deduced that the greater heavy metals content may be a reason for high phytotoxic activity of Ph.Chf. It is also revealed from the data that *P.hydropiper* could be a significant source of natural herbicides for weeds control in a sustainable manner to increase per acre yield.

Anthelmintic drugs are losing their efficacy due to resistance of different nematodes to these drugs especially as a single dose regimen [45]. Widespread efforts have been made to find out more effective and safe anthelmintic drugs. Plant extracts and crude saponins are potential sources of anthelmintic drugs and have been extensively studied for anthelmintic activities [21, 34]. Results of our anthelmintic investigation against *P. posthuma* and *A. galli* are mentioned in Figures 1(a,b) and 2(a,b). All fractions showed dose dependent response against the tested parasites. The data revealed that Ph.Sp, Ph.Bt, Ph.Chf and Ph.EtAc exhibited significant anthelmintic activity against *P. posthuma* at 10 mg/ml concentration. Whereas Ph.EtAc, Ph.Chf and Ph.Aq were most effective against *A. galli.* Anthelmintic activities of these fractions were comparable to positive control drugs (albendazole and levamisole HCl) at the same concentration.

## Conclusions

This study reveals that *P. hydropiper* contain Fe, Pb, Cu, Cd, Zn, Cr, Co, secondary metabolites like; alkaloids alkaloids, flavonoids, saponins, tannins, terpenoids, anthraquinones and glycosides. The plant crude extract, its various fractions and crude saponins exhibited concentration dependent phytotoxic and anthelmintic actions, and therefore may be excellent sources of phytotoxic and anthelmintic constituents that warrant their isolation and purification. Further investigations including activity guided isolation is in progress in our laboratory.

## References

[1] Khan S, Gul M (2007). In vitro antifungal activity of *Rhazya stricta*. Pak J Pharm Sci.

[2] Ahmad M, Sultana S, Hadi S, Hadda T, Rashid Zafar M, Khan M, Khan M, Yaseen G (2014). An ethnobotanical study of medicinal plants in high mountainous region of Chail valley (District Swat- Pakistan). J Ethnobiol Ethnomed.

[3] Abbasi A, Khana MA, Mushtaq A, Muhammad Z, Sarwat J, Shahzia S (2010). Ethnopharmacological application of medicinal plants to cure skin diseases and in folk cosmetics among the tribal communities of North-West Frontier Province. Ethnopharmacol.

[4] David J (2008). Mabberley’s Plant-Book Third Edition.

[5] Sharma R (2003). Medicinal Plants of India-An Encyclopedia.

[6] Chevallier A (1996). The Encyclopedia of Medicinal Plants.

[7] Wang K-W, Zhu J-R, Shen L-Q (2013). A new lignan with anti-tumour activity from *Polygonum perfoliatum* L. Nat Prod Res.

[8] Yang Y, Yu T, Jang H-J, Byeon SE, Song S-Y, Lee B-H, Rhee MH, Kim TW, Lee J, Hong S (2012). In vitro and in vivo anti-inflammatory activities of *Polygonum hydropiper* methanol extract. J Ethnopharmacol.

[9] S Redzic S (2007). The ecological aspect of ethnobotany and ethnopharmacology of population in Bosnia and Herzegovina. Collegium Antropologicum.

[10] Popovic Z, Smiljanic M, Kostićb M, Nikić P, Janković S (2014). Wild flora and its usage in traditional phytotherapy (Deliblato Sands, Serbia, South East Europe). Ind J Trad Knowl.

[11] Qureshi R, Ghufran M, Sultana K, Ashraf M, Khan A (2008). Ethnomedicinal studies of medicinal plants of Gilgit District and surrounding areas. Ethnobot Res Appl.

[12] Choudhary RK, Oh S, Lee J (2011). An ethnomedicinal inventory of knotweeds of Indian Himalaya. J Med Plants Res.

[13] Ayaz M, Junaid M, Ahmed J, Ullah F, Sadiq A, Ahmad S, Imran M (2014). Phenolic contents, antioxidant and anticholinesterase potentials of crude extract, subsequent fractions and crude saponins from *Polygonum hydropiper* L. BMC Complement Altern Med.

[14] Farrukh H, Ishfaq H, Ghulam D, Shams-un-Nisa, Ibrar K, Bashir A (2010). Cytotoxicity and phytotoxicity of some selected medicinal plants of the family Polygonaceae. Afr J Biotechnol.

[15] Hussain I, Khan F, Khan I, Khan L (2006). Determination of heavy metals in medicnal plants. J Chem Soc Pak.

[16] Khan SU, Khan A-u, Shah A-u-HA, Shah SM, Hussain S, Ayaz M, Ayaz S (2013). Heavy metals content, phytochemical composition, antimicrobial and insecticidal evaluation of *Elaeagnus angustifolia*. Toxicol Ind Health.

[17] Schimmer O, Haefele F, Kruger A (1988). The mutagenic potencies of plant extracts containing quercetin in *Salmonella typhimurium*TA98 and TA100. Mutat Res.

[18] Higashimoto M, Purintrapiban J, Kataoka K, Kinouchi T, Vinitketkumnuen U, Akimoto S, Matsumoto H, Ohnishi Y (1993). Mutagenicity and antimutagenicity of extracts of three species and a medicinal plant in Thailand. Mutat Res.

[19] Krishnaiah D, Devi T, Bono A, Sarbatly R (2009). Studies on phytochemical constituents of six Malaysian medicinal plants. J Med Plants Res.

[20] Price KR, Eagles J, Fenwick GR (1988). Saponin composition of 13 varieties of legume seed using fast atom bombardment mass spectrometry. J Sci Food Agric.

[21] Yuan C, Wang C, Wicks S, Qi L (2010). Chemical and pharmacological studies of saponins with a focus on American ginseng. J Ginseng Res.

[22] Lu X, Qiu S, Sun X, Li Z (2005). Preliminary study on the capability of antioxidation and scavenging free radicals of sasanquasaponins. Food Sci.

[23] Pal D, Sannigrahi S, Mazumder U (2009). Analgesic and anticonvulsant effects of saponin isolated from the leaves of *Clerodendrum infortunatum* Linn. in mice. Ind J Exp Biol.

[24] Seth R, Sarin R (2010). Analysis of the phytochemical content and anti-microbial activity of *Jatropha gossypifolia* L. Arch Appli Sci Res.

[25] Shah SM, Ayaz M, A-u K, Ullah F, Farhan, Shah A-u-HA, Iqbal H, Hussain S (2013). 1,1-Diphenyl,2-picrylhydrazyl free radical scavenging, bactericidal, fungicidal and leishmanicidal properties of *Teucrium stocksianum*. Toxicol Ind Health.

[26] Geary TG, Woo K, McCarthy JS, Mackenzie CD, Horton J, Prichard RK, de Silva NR, Olliaro PL, Lazdins-Helds JK, Engels DA (2010). Unresolved issues in anthelmintic pharmacology for helminthiases of humans. Int J Parasitol.

[27] Blumenthal DS, Schultz MG (1975). Incidence of intestinal obstruction in children infected with *Ascaris lumbricoides*. Am J Trop Med Hyg.

[28] Bethony J, Brooker S, Albonico M, Geiger SM, Loukas A, Diemert D, Hotez PJ (2006). Soil-transmitted helminth infections: ascariasis, trichuriasis, and hookworm. Lancet.

[29] Ivory C, Chadee K (2004). DNA vaccines: designing strategies against parasitic infections. Genet Vaccine Ther.

[30] Dhar D, Sharma R, Bansal G (1982). Gastro-intestinal nematodes in sheep in Kashmir. Vet Parasitol.

[31] Coles GC (1997). Nematode control practices and anthelmintic resistance on British sheep farms. Vet Rec.

[32] Liu LX, Weller P (1996). Antiparasitic drugs. N Engl J Med.

[33] Ali N, Shah S, Shah I, Ahmed G, Ghias M, Imran K (2001). Cytotoxic and anthelmintic potential of crude saponins isolated from *Achillea Wilhelmsii* C. Koch and *Teucrium Stocksianum* boiss. BMC Complement Altern Med.

[34] Akhtar M, Iqbal Z, Khan M, Lateef M, Bachman D (2000). Anthelmintic activity of medicinal plants with particular reference to their use in animals in the Indo-Pakistan subcontinent. Small Ruminant Res.

[35] Konan AB, Datte JY, Yapo P (2008). Nitric oxide pathway-mediated relaxant effect of aqueous sesame leaves extract (*Sesamum radiatum Schum. & Thonn*.) in the guinea-pig isolated aorta smooth muscle. BMC Complement Altern Med.

[36] Khan FA, Ullah Z, Haider S (2011). Phytochemicals screening and antimicrobial activities of selected medicinal plants of Khyberpakhtunkhwa Pakistan. Afr J Pharm Pharmacol.

[37] Khan SA, Khan L, Hussain I, Marwat KB, Akhtar N (2008). Profile of heavy metals in selected medicinal plants. Pak J Weed Sci Res.

[38] Meriga B, Mopuri R, Krishna T (2012). Insecticidal, antimicrobial and antioxidant activities of bulb extracts of *Allium sativum*. Asian Pac J Trop Med.

[39] Arzu U, Camper N (2002). Biological activity of common mullein, a medicinal plant. J Ethnopharm.

[40] Parida S, Patro VJ, Mishra US, Mohapatra L, Sannigrahi S (2010). Anthelmintic potentials of crude extracts and its various fractions of different parts of *Pterospermum Acerifolium* Linn. Inter J Pharm Sci Rev Res.

[41] McGrath SP, Smith S, Alloway BJ (1990). Chromium and nickel in heavy metals in soils. Blackie, Glasgow.

[42] Khuda F, Iqbal Z, Zakiullah, Khan A, Nasir F, N M (2012). Metal analysis, phytotoxic, insecticidal and cytotoxic activities of selected medicinal plants of Khyber Pakhtunkhwa. Pak J Pharm Sci.

[43] WHO (1989). WHO Technical Report Series 776. Evaluation of Certain Food Additives and Contaminants.

[44] Zeb A, Sadiq A, Ullah F, Ahmad S, Ayaz M (2014). Phytochemical and toxicological investigations of crude methanolic extracts, subsequent fractions and crude saponins of *Isodon rugosus*. Biol Res.

[45] Mccarthy J (2005). Is anthelmintic resistance a threat to the program to eliminate lymphatic filariasis. Am J Trop Med Hyg.

[CR46] The pre-publication history for this paper can be accessed here:http://www.biomedcentral.com/1472-6882/14/465/prepub

